# Motif statistics and spike correlations in neuronal networks

**DOI:** 10.1186/1471-2202-13-S1-P43

**Published:** 2012-07-16

**Authors:** Yu Hu, James Trousdale, Krĕsimir Josić, Eric Shea-Brown

**Affiliations:** 1 Department of Applied Mathematics, University of Washington, Seattle, WA 98195, USA; 2 Department of Mathematics, University of Houston, Houston, TX, 77204-5001, USA; 3 Department of Biology and Biochemistry, University of Houston, Houston, TX, 77204-5001, USA; 4 Program in Neurobiology and Behavior, University of Washington, Seattle, WA 98195, USA

## 

Motifs are patterns of subgraphs that are the building blocks of complex networks. Recent experiments have characterized the frequencies with which different motifs occur in biological neural networks, and found remarkable deviations from what we would expect if the networks were randomly connected [1]. Here, we study the impact of such patterns of connectivity on the level of correlated, or synchronized, spiking activity among pairs of cells. Correlations in spiking activity have been shown to strongly impact the neural coding of information.

We use a linear, stochastic model of recurrent networks. A cell’s time-dependent firing rate is perturbed from its baseline level by convolution of a response kernel and the input signal from presynaptic neurons. Each neuron generates spikes as an inhomogeneous Poisson process. Previous studies have shown that such models can capture pairwise correlations in integrate and fire networks [2,3], and they are closely related to spike response and Hawkes models [4,6].

**Figure 1:**
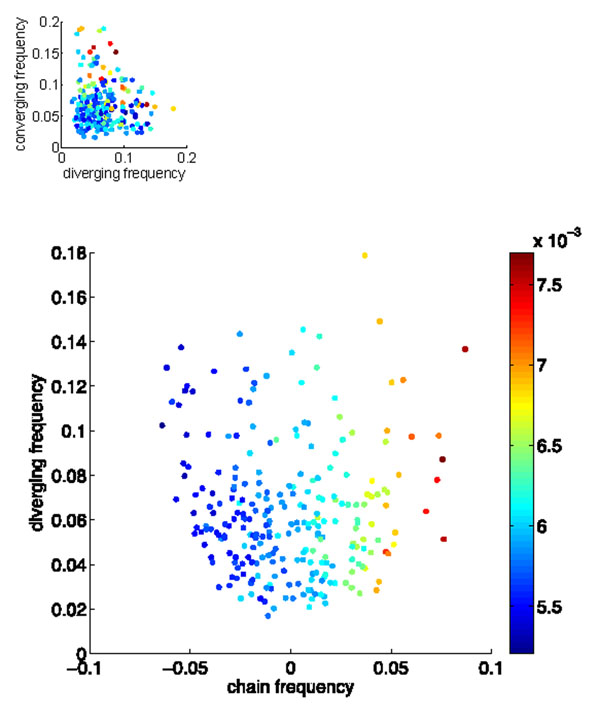
Each dot represent one network sample plotted against its chain and diverging motif frequencies. Color shows the standard deviation of correlations in the network. Inset is the same plot with respect to diverging and converging motifs.

For this model, there is an explicit expression for pairwise correlation in terms of the connectivity matrix. By expanding this expression in a series, one can relate each term to a different motif (with a different number of connections). Through a resumming technique, we show that the average correlation across the network can be closely approximated using the frequencies of only first and second order motifs. These are the diverging motif—two cells both receiving projections from another cell, its counterpart the converging motif—two cells projecting to a common cell, and the chain motif—three cells linked by two consecutive projections. Specifically, we show that the prevalence of diverging and chain motifs tends to increase correlation, while the converging motif makes no contribution to the average correlation. Moreover, we numerically show that *variance* of correlations across the network is largely determined by the frequency of the chain motif (see Figure 1) alone. Finally, we demonstrate potential effect of motif statistics on neural coding by showing how motif frequencies impact linear Fisher information. In particular, we find that the linear Fisher information is only affected by converging motif frequency (more information with given more converging motifs).
